# Inverse Molecular Docking as a Novel Approach to Study Anticarcinogenic and Anti-Neuroinflammatory Effects of Curcumin

**DOI:** 10.3390/molecules23123351

**Published:** 2018-12-18

**Authors:** Veronika Furlan, Janez Konc, Urban Bren

**Affiliations:** 1 Faculty of Chemistry and Chemical Technology, University of Maribor, Smetanova 17, SI-2000 Maribor, Slovenia; veronika.furlan@um.si; 2 National Institute of Chemistry, Hajdrihova 19, SI-1000 Ljubljana, Slovenia

**Keywords:** curcumin, anticarcinogenic effects, anti-neuroinflammatory effects, mechanistic insights, inverse molecular docking

## Abstract

Research efforts are placing an ever increasing emphasis on identifying signal transduction pathways related to the chemopreventive activity of curcumin. Its anticarcinogenic effects are presumably mediated by the regulation of signaling cascades, including nuclear factor κB (NF-κB), activator protein 1 (AP-1), and mitogen-activated protein kinases (MAPK). By modulating signal transduction pathways, curcumin induces apoptosis in malignant cells, thus inhibiting cancer development and progression. Due to the lack of mechanistic insight in the scientific literature, we developed a novel inverse molecular docking protocol based on the CANDOCK algorithm. For the first time, we performed inverse molecular docking of curcumin into a collection of 13,553 available human protein structures from the Protein Data Bank resulting in prioritized target proteins of curcumin. Our predictions were in agreement with the scientific literature and confirmed that curcumin binds to folate receptor β, DNA (cytosine-5)-methyltransferase 3A, metalloproteinase-2, mitogen-activated protein kinase 9, epidermal growth factor receptor and apoptosis-inducing factor 1. We also identified new potential protein targets of curcumin, namely deoxycytidine kinase, NAD-dependent protein deacetylase sirtuin-1 and -2, ecto-5′-nucleotidase, core histone macro-H2A.1, tyrosine-protein phosphatase non-receptor type 11, macrophage colony-stimulating factor 1 receptor, GTPase HRas, aflatoxin B1 aldehyde reductase member 3, aldo-keto reductase family 1 member C3, amiloride-sensitive amine oxidase, death-associated protein kinase 2 and tryptophan-tRNA ligase, that may all play a crucial role in its observed anticancer effects. Moreover, our inverse docking results showed that curcumin potentially binds also to the proteins cAMP-specific 3′,5′-cyclic phosphodiesterase 4D and 17-β-hydroxysteroid dehydrogenase type 10, which provides a new explanation for its efficiency in the treatment of Alzheimer’s disease. We firmly believe that our computational results will complement and direct future experimental studies on curcumin’s anticancer activity as well as on its therapeutic effects against Alzheimer’s disease.

## 1. Introduction

With the availability of an ever increasing number of three-dimensional protein structures and the onset of high-performance computing systems, molecular docking provides a fast, low-cost alternative to the experimental screening of large compound libraries [1]. In molecular docking, many small molecules are typically docked into a given protein and their binding free energies are estimated with the aim to reduce the time and effort required to identify new candidate drug molecules for further development [2]. The calculation of the binding free energies is simplified using various assumptions and estimated by a value also known as “score” given to the docked binding conformations [3]. However, a small molecule drug may interact with many other proteins (off-targets), which can have significant impacts on drug’s overall biological activity, efficacy, promiscuity, and side-effects. In inverse docking, a single small molecule is docked into a collection of protein structures enabling early prediction of a drugs side-effects, as well as toxicity. Inverse docking therefore plays an important role in modern drug discovery and design.

**Figure 1:**
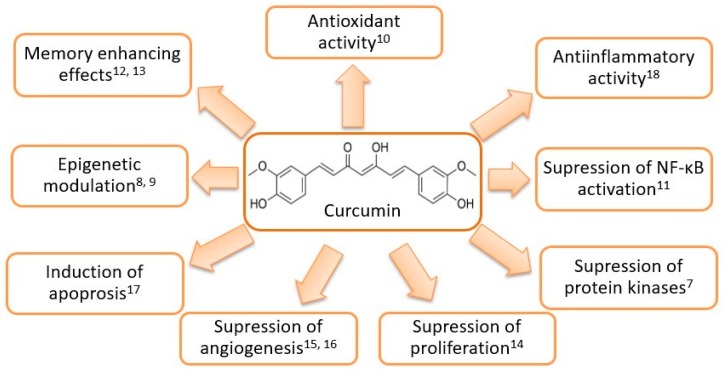
Structural formula and reported biological effects of curcumin [7,8,9,10,11,12,13,14,15,16,17,18].

Curcumin (diferuloylmethane) is derived from the rhizome of the plant *Curcuma longa* (*Zingiberaceae* family) native to Southeast Asia and represents the main component of the turmeric spice [4]. *C. longa* contains a class of compounds known as curcuminoids, namely curcumin, demethoxycurcumin, and bisdemethoxycurcumin [5]. Curcuminoids consist of two methoxylated phenolic groups connected by two α,β unsaturated carbonyl groups that exist in an enol form at physiological pH [6]. As the main curcuminoid, curcumin comprises approximately 2% to 5% of the turmeric spice and is largely responsible both for its yellow color and its therapeutic effects [4]. Curcumin is known to possess antioxidant, antiseptic, analgetic, antimicrobial, anti-inflammatory as well as anticarcinogenic properties and is considered pharmacologically safe [4]. Reported biological activities of curcumin are presented in Figure 1.

### 1.1. Anticarcinogenic, Antioxidant and Anti-Inflammatory Properties of Curcumin 

Curcumin was found to prevent inflammatory diseases, such as the bowel disease, pancreatitis, arthritis, and chronic anterior uveitis [5]. Inhibition of carcinogenesis was demonstrated by in vivo and in vitro studies at all three stages: Tumor initiation, tumor promotion (proliferation), and progression (angiogenesis and formation of metastases) [19]. Curcumin also possesses a strong antioxidant activity and acts as a potent scavenger of a variety of reactive oxygen and nitrogen species including superoxide anion radicals, hydroxyl radicals [20] as well as nitrogen dioxide radicals [21]. Additionally, it was demonstrated that reduced ^•^NO generation following curcumin treatment may relieve neuroinflammation associated with degenerative conditions such as Alzheimer’s disease [22]. However, degradation products of curcumin might also be responsible for its observed biological effects [23].

Due to its lipophilicity, curcumin is able to cross the nuclear membrane and take part in epigenetic regulation [24]. Curcumin was reported to target chromatin-modifying enzymes histone acetyltransferases (HATs) and histone deacetylases (HDACs). The balance between acetylation and deacetylation of histone proteins indeed plays a crucial role in the regulation of gene expression [12]. Curcumin reduces histone acetylation via inhibition of HAT activity and therefore inhibits gene expression [9]. It was shown to reduce HDAC activity and directly inhibit the transcription of the gene HDAC4 in medulloblastoma [10]. Curcumin also influences the activity of methyltransferases and demethyltransferases. It favors demethylation at hypermethylated regions of tumor suppressor genes and methylation of oncogenes, which are over-expressed in most cancers [12].

Modifications in cellular signaling pathways that regulate cell proliferation have been associated with carcinogenesis. For instance, the abnormal activation of mitogen-activated protein kinases (MAPK) pathway, which results in uncontrolled cell proliferation, was reported in several types of cancer [15]. Curcumin was also shown to inhibit nuclear factor κB (NF-κB), a transcription factor involved in the inflammatory and innate immune responses as well as in cellular responses to stress [12]. Nuclear factor κB activates proliferative genes through interaction with specific proteins associated with inhibition of phosphorylation of NF-κB inhibitory protein IκB [25]. During proliferation, curcumin also down-regulates the formation of proinflammatory cytokines, such as tumor necrosis factor α (TNF-α) and interleukin 1 (IL-1) [26]. Activator protein 1 (AP-1) is another transcription factor that regulates the expression of genes involved in cellular proliferation in a variety of tumors including glioblastoma, cervical cancer, breast cancer, as well as in head and neck cancer [12]. According to Rashmi et al. [27] curcumin is able to inhibit AP-1 activation. Curcumin was also shown to downregulate the β-catenin and the related wingless-related integration site (Wnt) signaling pathway, which results in the nuclear translocation of β-catenin and in activation of target genes regulating cell proliferation, differentiation, and survival [12,28].

Moreover, curcumin may block carcinogenesis through the induction of apoptosis (programmed cell death). There are two main apoptotic pathways: The intrinsic (mitochondrial) pathway and the extrinsic (death receptor) pathway [29]. Curcumin has been shown to selectively induce apoptosis in tumor cells via the upregulation of p53 expression [30] and the initiation of the mitochondrial apoptotic pathway by an increased Bax gene expression and cytochrome c release [31]. Curcumin also exhibits a stimulatory effect on the extrinsic apoptotic pathway, which is triggered by the binding of TNF-α and Fas ligand to their corresponding cell surface receptors. Their activation results in stimulation of caspase-8 via the receptor-attached fas-associated protein with death domain (FADD) adapter molecule and initiation of the caspase cascade which induces apoptosis [29]. Furthermore, Kim et al. [32] reported curcumin’s cytotoxic activity involving reactive oxygen species (ROS) in oral squamous cell carcinoma via induction of apoptosis. 

At the final phase of carcinogenesis, the progression, curcumin exerts beneficial effects by the inhibition of angiogenesis. Angiogenesis represents the growth of new blood vessels from pre-existing vessels and it is also a fundamental step in tumor progression from a benign to a malignant or invasive state. Cancer cells can, therefore, dissociate from the main tumor and be carried through the circulation to distant tissues where they implant and initiate formation of secondary tumors (metastases) [12]. Curcumin was found to suppress malignant cell migration, invasion, and formation of metastases in vivo, which has been mainly related to its ability to downregulate the matrix metalloproteinases (MMPs), namely, MMP-2 and MMP-9 [33].

### 1.2. Curcumin and Alzheimer’s Disease

Alzheimer’s disease (AD) is a neurodegenerative disorder characterized by the accumulation of β-amyloid peptides (Aβ) and neurofibrillary tangles (NFTs) in the brain, by the widespread cortical neuronal loss and progressive memory impairment (progressive dementia) [34]. A growing amount of evidence indicates that oxidative stress, free radicals, β-amyloid, cerebral deregulation caused by bio-metal toxicity and abnormal inflammatory processes contribute to the AD pathology. The accumulation of Aβ 40 as well as Aβ 42 and their deposition in insoluble plaques are the major neuropathological hallmarks of the AD. However, specific mechanisms causing AD and memory deficits remain unknown due to a lack of scientifically proven pharmacological strategies [35].

Dietary supplementation with curcumin may improve the cognitive functions of patients with neurodegenerative diseases such as AD [13,14]. Due to various effects of curcumin, such as decreased formation of β-amyloid plaques, delayed degradation of neurons, metal-chelation, anti-inflammatory, antioxidant and decreased microglia formation, the overall memory of patients with AD has improved [36]. However, it remains unknown whether the memory-enhancing effects of curcumin on cognitive disorders associated with the AD is related to the inhibition of phosphodiesterase enzymes (PDEs), which results in an increase of intracellular cyclic adenosine monophosphate (cAMP) and/or cyclic guanosine monophosphate (cGMP) activities.

In this paper, we report an inverse molecular docking of curcumin against human target proteins, resulting in protein targets to which curcumin potentially binds. In our inverse molecular docking approach, curcumin is docked into the active sites of all available human protein structures using a novel computer algorithm CANDOCK [37], which accounts for protein flexibility. The hierarchical nature of this method is derived from an ‘atoms to fragments,’ ‘fragments to ligands’ approach that generates all chemically relevant poses of a given ligand at the surrounding protein binding site. We firmly believe that our results represent a strong basis for novel experimental studies on anticarcinogenic activity of curcumin as they provide new plausible molecular mechanisms for its well-established anticarcinogenic effects. Moreover, our findings provide a new possible explanation for curcumin’s efficiency in the treatment of AD. The established protocol can, therefore, guide new experimental studies on effective treatment of AD and several types of cancer using curcumin.

## 2. Computational Methods

### 2.1. Inverse Molecular Docking of Curcumin into Human Proteins

Inverse molecular docking was performed with the fragment-based CANDOCK program [37], which was used to screen curcumin against all available human protein structures obtained from the Protein Data Bank (PDB) [38]. As blind docking was unfeasible due to the size of the PDB, we identified small molecule binding sites on the proteins and prepared them for inverse docking according to the procedure described in [39]. A binding site was defined as the space occupied by the union of one to several ligands that bind to similar binding sites and for which a co-crystal structure exists in the PDB. The docking search space was therefore focused on binding sites, which reduced the time complexity of the inverse docking. A previous version of this database was successfully used for discovery of small-molecule inhibitors of inhibin α (InhA) enzyme in *Mycobacterium tuberculosis* and this resulted in the identification of three previously unrecognized inhibitors with novel scaffolds [39].

#### 2.1.1. Proteome-Wide Binding Site Preparation

Currently, we have updated the small molecule binding site database [39] to include newly released protein structures. Here we summarize the key steps taken to update the database:

Step 1: For each protein chain (PDB/Chain identifier) the presumed biological structural form was constructed using the data in the header of the corresponding PDB file. Next, the co-crystallized ligands of the protein chain identified by HETATM records with >7 heavy atoms, where at least one atom is <3 Å away from the particular protein chain, were considered. From each extracted ligand we determined binding residues, i.e., protein atoms <5 Å away from any ligand atoms. Finally, binding sites surface files were generated. 

Step 2: The ~310,000 protein chains in the PDB were clustered using a sequence identify cutoff at 100%, resulting in 70,000 protein chain clusters. The binding sites obtained in Step 1 were then assigned to their associate sequence clusters. Each binding site was assigned to a cluster that contains the protein (identified by PDB/Chain identifier) from which this binding site was extracted. We did not consider water molecules and cofactors inside the binding pocket, which were removed prior to the inverse molecular docking simulation.

Step 3: Each pair of binding sites was compared in an all-against-all fashion within each 100% sequence identity cluster using the ProBiS algorithm. The ProBiS web server algorithm enables the detection of structurally similar protein binding sites as well as the local pairwise alignment of X-ray or NMR determined protein structures from the PDB [40].

Step 4: The binding sites within each 100% sequence identity cluster were then clustered. In this second clustering the similar binding site pairs (with z-score ≥ 2.0) were assigned to the same cluster. Such clustering of binding sites within existing sequence identity clusters ensures that if a protein has two or more different binding sites, then each one can be assigned to a separate binding site cluster.

Step 5: The representative binding site surfaces (about 35,000 surfaces) together with the co-crystallized ligands transposed to the representative binding sites were chosen.

Step 6: The pre-calculated database of binding sites and ligands was then prepared for each existing protein structural chain in the PDB (~310,000) by comparing that particular chain with the binding site surfaces using the ProBiS algorithm (Step 5). The identified structural similarities allowed for feasible predictions of binding sites for (even non-representative) protein chains for which binding sites have not been identified yet. Binding sites were defined as the union of centroids, i.e., the entire space that multiple spheres occupy, that follow the contours of the binding site.

Step 7: We then filtered the obtained small molecule ligand binding sites database for the human proteins, resulting in a total of 13,553 small molecule binding sites on human proteins, which were used as the input for inverse molecular docking with the newly developed CANDOCK program.

#### 2.1.2. CANDOCK Docking Algorithm

The CANDOCK docking algorithm employs a hierarchical approach to reconstruct small molecule ligands from an atomic grid using graph theory [41] and a generalized statistical potential function. The algorithm generates a docked ligand hierarchically by docking ligand fragments in a protein’s binding pocket using an atomic grid and a pair-wise knowledge-based scoring to select the best-docked poses of the ligand fragments. These ligand fragments are linked together using the fast maximum clique algorithm [41] to create a large number of chemically relevant ligand conformations in the target protein leading to a thorough sampling of the ligand’s conformational space. Energy minimization procedures are used to model the flexibility of the small ligand and the protein during the docking procedure [37].

The docking scores of the ligand, in this case the curcumin, to its potential target proteins were calculated using the radial mean distribution scoring function RMR6 [37]. The knowledge-based docking score has arbitrary units (arb. units) and represents relative binding free energy of the curcumin to a given protein. As the crystal structures of curcumin bound to the predicted protein targets have not been experimentally determined yet, the docked conformation with the lowest docking score value was considered as the most likely binding conformation of curcumin.

The inputs to the CANDOCK algorithm were the curcumin structure to be inversely docked as well as the set of human protein structures with their binding sites’ locations defined as multiple centroids. The output was a ranked list of the docked and minimized protein-curcumin complex structures.

### 2.2. Distribution of Docking Scores

**Figure 2:**
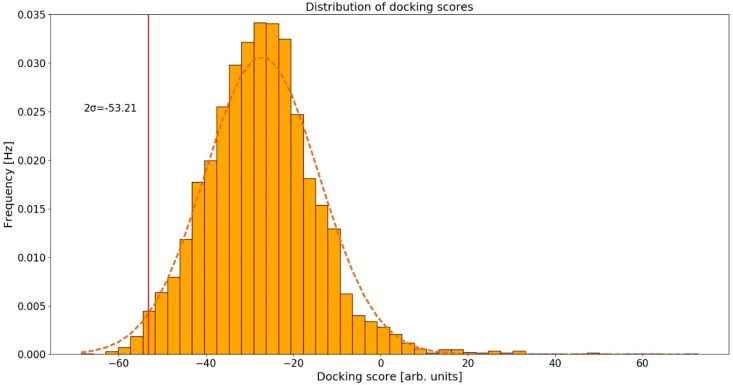
Normal distribution fitting of calculated docking scores.

The calculated conformations of curcumin in human protein binding sites were ranked according to their docking scores. We assumed that the docking scores were normally distributed based on the normal Q-Q plot [42], and the corresponding 95% confidence interval was (−53.21, −1.73) arb. units (Figure 2). As potential curcumin targets, we considered those proteins whose docking scores were below the 95% confidence interval, that is <−53.21 arb. units. By setting the threshold at 95% we are considering only the top-scoring curcumin-protein complexes. Proteins that have scores below this threshold represent the ones most likely to be the actual targets of curcumin. This threshold allowed us to obtain a reasonably large, but still manageable, set of proteins to inspect manually as only the top-ranked protein targets were selected as candidates for further experimental assays and were therefore examined in detail.

### 2.3. Validation of Inverse Docking Methodology

The aim of the inverse molecular docking methodology with a given ligand and a set of proteins is to provide a ranking of proteins that prioritizes the actual protein targets of this ligand over proteins that do not bind this ligand [43,44]. In contrast to the classical virtual screening, where multiple potential ligands are screened against a single protein, this inverse process results in a list of potential protein targets for the ligand, ranked according to their predicted affinity (docking scores) towards the ligand. Receiver operating characteristics (ROC) curves [44], enrichment curves [45], and the recently introduced Predictiveness Curves (PC) [46] provide intuitive visualization of the inverse docking results. The ROC metric is a chart with the true positive fraction (TPF) on the y-axis versus the false positive fraction (FPF) on the x-axis for each protein in the list ranked by the docking scores. Here, true positive targets correspond to the experimentally confirmed protein targets of curcumin found in the ChEMBL database, while false positives are those targets that were predicted to bind curcumin, but have not been experimentally confirmed yet. Each point on a ROC curve represents a unique FPF/TPF pair corresponding to a particular fraction of the protein set, which allows estimation of the inverse docking method’s ability to discriminate true positive protein targets from false positive protein targets [44]. Enrichment curves allow the quantification of the early recognition of target proteins by visualizing the TPF on the y-axis for each fraction of the ordered protein set fraction on a logarithmic scale (x-axis) [45]. Predictiveness curves (PC) also allow quantification of the early recognition of target proteins by visualizing the TPF (y-axis) for each fraction of the ordered data set (x-axis). A generalized linear model is built to issue probabilities of activity (y-axis) for each compound of the ordered dataset (x-axis), as a function of the scores and the rank of true positive protein targets. Moreover, PC allows detection of potential score gaps and variations issued by a scoring function in the detection of the actives, which correspond to gaps in probabilities of activity [46]. The area under the ROC curve (ROC AUC) accounts for the overall discrimination of the true target proteins while the Boltzmann enhanced discrimination of ROC (BEDROC) [45], the enrichment factor value at 1% of screened compounds (EF) and the robust initial enhancement (RIE) [47] aim to quantify their early recognition. The standardized total gain (TG), calculated from the PC, summarizes the discrimination of true protein targets imputable to the score variation over a complete protein set [46]. TG values over 0.25 combined with a ROC AUC over 0.5 generally signify that score variations are relevant in the discrimination of the actives. High TG values (over 0.4) combined with a ROC AUC over 0.5 therefore indicate that the screening method performed well and that its performance should be reproducible in similar experimental conditions [46]. We used a web-based interactive application Screening explorer [48], that covers all the aspects of the analysis of the results, to evaluate the performance of our newly developed inverse molecular docking protocol.

## 3. Results and Discussion

### 3.1. Identified Protein Targets

We identified 21 potential curcumin protein targets using the docking score threshold of −53.21 arb. units (see Computational methods). Their docking scores, functions and reported connections with known diseases are presented in Table 1.

We only found the IC_50_ value for direct interaction between AP-1 and curcumin (6.9 nM) [76], based on which we could not conclude on the correlation with the docking scores.

In the following subsections, we describe the novel potential protein targets of curcumin in detail and put our findings in the context with the current knowledge.

### 3.2. Anticarcinogenic Effects of Curcumin Explained by the Identified Protein Targets

We identified NAD-dependent protein deacetylase sirtuin-2, core histone macro-H2A.1, and NAD-dependent protein deacetylase sirtuin-1 as potential targets of curcumin (Table 1). Curcumin was already reported to target chromatin-modifying enzymes histone deacetylases (HDACs). It was shown to reduce HDAC activity and to directly inhibit the transcription of gene HDAC4 in medulloblastoma [10]. The balance between acetylation and deacetylation of histone proteins plays a crucial role in the regulation of gene expression [12]. However, the connection between specific chromatin-modifying enzymes and curcumin remains unknown. The identified enzymes, therefore, provide a possible explanation for the observed epigenetic modulation of curcumin, resulting in a repressed transcription by deacetylation of histones.

We also identified DNA (cytosine-5)-methyltransferase 3A as a potential curcumin target (Table 1), which is in agreement with Zamani et al. [56], who already reported that curcumin influences the activity of methyltransferases. This protein target thus supports and complements the finding that curcumin favors the methylation of oncogenes, which are overly expressed in most cancers, and thus actively represses their transcription. 

Moreover, we identified MMP-2 and MAPK-9 as potential protein targets of curcumin, which is in agreement with Su et al. [59] and Yu et al. [64], who already reported the interaction of curcumin with MMP-2 and MAPK-9. According to the literature, the abnormal activation of the mitogen-activated protein kinases (MAPK) pathway results in an uncontrolled cell proliferation and has been observed in several types of cancer [15]. Furthermore, we identified new potential protein targets of curcumin connected with cell proliferation, namely macrophage colony-stimulating factor 1 receptor, aldo-keto reductase family 1 member C3, amiloride-sensitive amine oxidase and tyrosine-protein phosphatase non-receptor type 11. These results, therefore, provide a new explanation for the already recognized inhibition of proliferation by curcumin. 

Using the inverse molecular docking protocol, we identified several proteins, namely MMP-2, NAD-dependent protein deacetylase sirtuin-2, core histone macro-H2A.1, NAD-dependent protein deacetylase sirtuin-1 and epidermal growth factor receptor, to which curcumin potentially binds and therefore regulates the activity of NF-κB. Transcription factor NF-κB is active in many cancer cells since it regulates the expression of genes linked to cell survival and proliferation. It is activated by and regulates the expression of proinflammatory cytokines such as TNF-α and IL-1 and, therefore, represents a key molecule in the regulation of inflammation [12]. Curcumin was already reported to inhibit NF-κB activation of proliferative genes through the interaction with specific proteins associated with the inhibition of phosphorylation of NF-κB inhibitory protein IκB [25] and the identified proteins provide a possible mechanistic explanation for the observed effects.

We also identified activator protein 1 (AP-1) as a potential target of curcumin, which provides another possible explanation of curcumin’s antiproliferative effects. AP-1 represents a transcription factor that regulates expression of genes involved in cellular proliferation in a variety of tumors, including glioblastoma, cervical cancer, breast cancer, as well as head and neck cancer [12]. These findings are in agreement with Rashmi et al. [27], who reported an interaction between curcumin and AP-1. Moreover, curcumin was already shown to inhibit AP-1 activation through a direct interaction with the AP-1 DNA-binding motif [85].

The obtained results also suggest that curcumin potentially regulates the activation of the Wnt/β-catenin pathway by binding to the MAPK-9 protein. Abnormal activation of the Wnt signaling pathway by β-catenin results in an abnormal activation of genes regulating cell proliferation, differentiation, cell growth and survival connected to cancer [12]. These findings are in agreement with Leow et al. [86], who reported that curcumin significantly inhibited the Wnt/β-catenin signaling and reversed the Wnt/β-catenin-induced cell invasiveness as well as MMP-9 expression in human osteosarcoma cells. 

We also identified new proteins to which curcumin potentially binds and thus regulates apoptosis (programmed cell death) of cancer cells: NAD-dependent protein deacetylase sirtuin-1, amiloride-sensitive amine oxidase and death-associated protein kinase 2. The obtained results also suggest curcumin’s suppression of the NF-κB and AP-1 mediated cell survival pathway as a possible mechanism of the compound’s pro-apoptotic effect [29]. Curcumin has been also shown to selectively induce apoptosis in tumor cells via the upregulation of p53 expression [30]. Our results indeed identified the possible connection between curcumin’s binding to NAD-dependent protein deacetylase sirtuin-1 and the upregulation of p53 expression.

Furthermore, we identified potential protein targets of curcumin, namely MMP-2 and tryptophan-tRNA ligase, which provide a new possible explanation for the observed regulation of angiogenesis by curcumin. Curcumin was already demonstrated to possess direct antiangiogenic activity in vitro [16] and in vivo [17] and can, therefore, at the final phase of carcinogenesis (progression), exert its beneficial effects by interruption of angiogenesis, the fundamental step in the progression of the tumor from a benign to a malignant or invasive state.

The MMP-2 and MMP-9 were also identified as potential targets of curcumin. These results are in agreement with Bachmeier et al. [33] who found that curcumin suppressed formation of metastasis in vivo and related the observed inhibitory effect to its ability to downregulate the MMPs, namely, MMP-2 and MMP-9. Our findings, therefore, provide a possible explanation for the observed in vivo suppression of malignant cell migration, invasion, and formation of metastasis by curcumin. Protein targets folate receptor β (FR-β) and cAMP-specific 3′,5′-cyclic phosphodiesterase 4D (PDE4D) with the lowest docking score values are described in detail in the following subsection.

### 3.3. Detailed Binding Poses of Curcumin in the Protein Targets FR-β and PDE4D with the Lowest Docking Score Values

**Figure 3:**
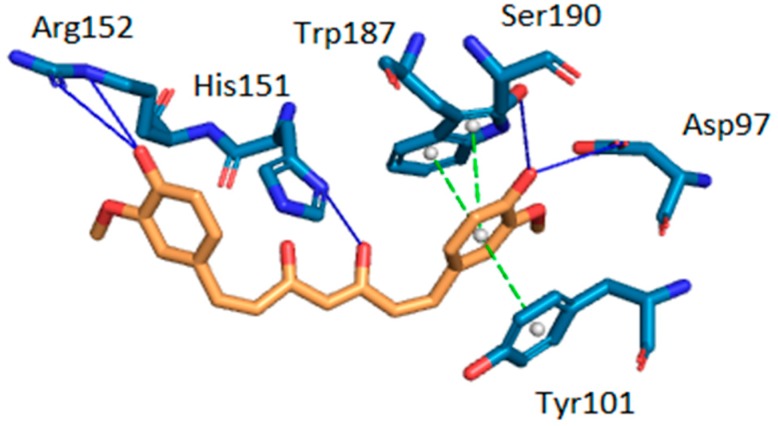
Intermolecular interactions between curcumin and the binding site of human folate receptor β (FR-β). Carbon atoms of curcumin are shown in orange and carbon atoms of amino-acid residues in light blue color. Oxygen atoms are red and nitrogen atoms dark blue. Hydrogen bonds are depicted in dark blue, π-stacking interactions in green. Hydrogen atoms are omitted for reasons of clarity.

**Figure 4:**
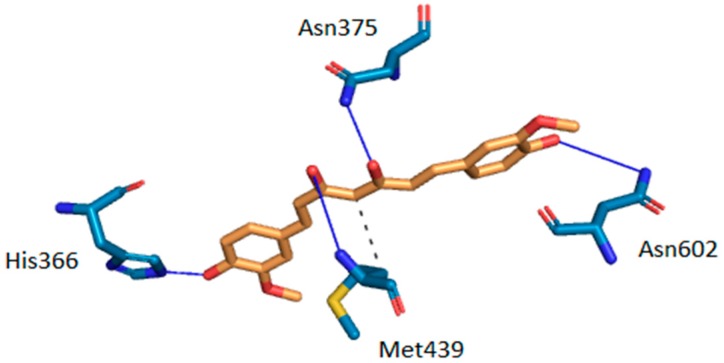
Intermolecular interactions between curcumin and the binding site of cAMP-specific 3′,5′-cyclic phosphodiesterase 4D (PDE4D). Carbon atoms of curcumin are shown in orange and carbon atoms of amino-acid residues in light blue color. Oxygen atoms are red, nitrogen atoms dark blue and sulfur atoms yellow. Hydrogen bonds are depicted in dark blue and hydrophobic interactions in gray color. Hydrogen atoms are omitted for reasons of clarity.

Intermolecular interactions between curcumin and the two target proteins with the lowest docking scores-folate receptor β (FR-β; PDB ID: 4kmy) and cAMP-specific 3′,5′-cyclic phosphodiesterase 4D (PDE4D; PDB ID: 3iad) are presented. The protein crystal structures of the FR-β and PDE4D proteins were obtained from the nr-PDB database [40] and the possible conformations of curcumin at their binding sites were generated using the CANDOCK docking program. The binding between the two proteins and curcumin was visualized by the Protein-Ligand Interaction Profiler (PLIP), which returns a list of detected intermolecular interactions of seven different types: Hydrogen bonds, hydrophobic contacts, π-stacking, π-cation interactions, salt bridges, water bridges and halogen bonds [87]. The highest ranked poses of curcumin at the binding sites of FR-β and PDE4D are shown in Figure 3 and Figure 4, respectively.

The docking results suggest that curcumin forms H-bonds with amino-acid residues Asp-97, Ser-190, Arg-152 and His-151 at the active site of FR-β through its phenol groups and linker chain hydroxyl group (Figure 3). On the other hand, it interacts with amino-acid residues His-336, Asn-375, Met-439, and Asn-602 at the active site of PDE4D through H-bonds, while residue Met-439 also forms a hydrophobic interaction (Figure 4). Curcumin may also form π-stacking interactions with Tyr-101 and double π-stacking interactions with Trp-187 at the active site of FR-β. The obtained results suggest that curcumin possesses a stronger binding affinity to FR-β (docking score of −63.30 arb. units) than to PDE4D (docking score of −62.24 arb. units), which nicely correlates with the number of detected intermolecular interactions. Further details about predicted intermolecular interactions are available in the Supplementary Materials.

### 3.4. Validation of the Inverse Molecular Docking Protocol

**Figure 5:**
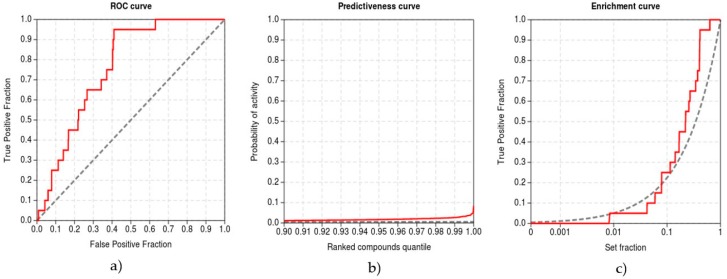
Validation of the inverse molecular docking protocol of curcumin against all human proteins from the Protein Data Bank: (**a**) the receiver operating characteristics (ROC) curve; (**b**) the predictiveness curve; and (**c**) the enrichment curve.

To validate the protocol, we performed inverse molecular docking using the CANDOCK program against a set of 13,553 human protein structures including 21 confirmed protein targets of curcumin whose measured IC50 values for curcumin were <10 µM [88]. We evaluated the ability of CANDOCK to discriminate the confirmed protein targets of curcumin from the non-target proteins using the established metrics (see Computational methods), which are shown in Figure 5.

Our inverse molecular docking protocol was successful in the discrimination of true target proteins from the non-targets with the ROC AUC of 0.932. The early recognition of protein targets was satisfactory, with the BEDROC of 0.370, RIE of 7.288, and EF1% of 4.745. The method produced meaningful score variations in the detection of true target proteins (TG 0.684), indicating that the developed protocol is expected to yield a good agreement with experiments.

### 3.5. Critical Perspective

We propose that curcumin possesses a potential as an antifolate drug and may provide some level of improvement in patients with overexpressed levels of the folate receptor β. This is in agreement with Dhanasekaran et al. [50] who found that curcumin modulates the uptake and cytotoxicity of methotrexate (MTX), an important antifolate chemotherapeutic, in KG-1 leukemic cells. Curcumin caused a significant, dose-dependent increase in the uptake of radiolabeled folic acid and MTX into KG-1 leukemic cells using a folate receptor β-targeted drug delivery system. The mechanism of curcumin action involves up-regulation of folate receptor β mRNA and FR-β protein in KG-1 leukemic cells. Our results imply that the mechanism behind this upregulation could be antagonistic activity of curcumin at the folate receptor β binding site, resulting in the induction of folate receptor β expression in KG-1 leukemic cells. Moreover, pre-treatment of KG-1 leukemic cells with non-toxic concentrations of curcumin significantly increased the cytotoxicity of MTX. Therefore, a combination of an optimum non-toxic dose of curcumin with a reduced concentration of MTX could potentially kill tumor cells, while reducing side effects associated with MTX treatment.

Folate receptor (FR), also known as the high-affinity folate-binding protein, represents a glycosylphosphatidylinositol (GPI)-linked membrane glycoprotein with a molecular weight of 38–40 kDa [89]. Two membrane-bound isoforms of FR, namely FR-α and FR-β, have been identified in humans [90]. While the receptor is generally absent from a majority of normal tissues, an elevated expression of FR has frequently been observed in various types of human cancer [91]. The identified protein target of curcumin with the lowest docking score FR-β, which shares ~70% sequence homology with FR-α, is frequently overexpressed in non-epithelial lineaged tumors, such as sarcomas and acute myeloid leukemia, as well as in activated macrophages associated with inflammation and malignant tumors [92,93,94]. Consequently, FR-β could serve as a prognostic marker for acute myeloid leukemia, for chronic inflammatory diseases such as rheumatoid arthritis, and for tumor-associated macrophages [92,93,94,95]. FR-β, therefore, plays an important role in cancer development, progression, and metastasis and is emerging as an effective therapeutic target in personalized medicine for treating of malignant and non-malignant cancers [94].

The recent availability of crystal structures of FR-α and FR-β in complex with folates and antifolates forms the basis for the design and the implementation of novel FR-targeted drugs for the treatment of cancer and inflammatory disorders [94]. However, drug discovery of novel antifolates with improved activities and fewer side effects remains an attractive research field for both academia and the pharmaceutical industry [90]. We suggest, that using a proper formulation (as nanoparticles [96,97] or liposomes [98]), curcumin drugs could be taken up by target tumor cells with expressed FR-β, in which they would exert their cytotoxic activity. To complement the current knowledge, we also provide detailed interactions of curcumin with specific amino-acid residues at the active site of FR-β. Our results, therefore, provide a detailed mechanistic insight into curcumin’s action as a potential antifolate drug. Based on the presented data, we believe that curcumin could provide an effective and non-toxic treatment in patients with overexpressed FR-β. Therefore, we suggest further experimental research in this direction. 

Using our inverse molecular docking protocol, we also identified a specific target protein, namely cAMP-specific 3′,5′-cyclic phosphodiesterase 4D (PDE4D), belonging to the cyclic nucleotide phosphodiesterase 4 (PDE4) family, to which curcumin potentially binds. The obtained results are in agreement with Abusnina et al. [52] who attributed the cancer-preventive and therapeutic effects of curcumin to PDE4D inhibition. To complement these findings, we provide detailed information about interactions of curcumin with the specific amino acid residues at the active site of PDE4D. Our data, therefore, yield a detailed mechanistic insight into curcumin’s binding affinity to PDE4D.

Moreover, the described connection with PDE4D also provides a potential explanation of curcumin’s efficiency in treating Alzheimer’s disease. PDE4D is a cAMP-specific enzyme, which is mainly distributed in inflammatory and immune cells and has been used as a therapeutic target for several diseases, such as asthma and chronic obstructive pulmonary disease (COPD) [99]. PDE4D might also be a potential target for treating metabolic diseases associated with aging, such as diabetes as well as cognitive impairment (dementia), neuroinflammatory and apoptotic processes in AD [35]. Recent studies suggest that the naturally occurring polyphenol curcumin inhibits aging-related pro-inflammatory cytokines expression, such as TNF-α and IL-1β, by increasing cyclic adenosine-3′, 5′ monophosphate (cAMP) levels [100,101]. The inhibition of PDE4D should consequently result in increased cAMP levels, which reportedly improve memory performance and decrease neuroinflammation and apoptosis [35]. Curcumin may, therefore, inhibit cAMP-specific PDE4D, and the related signaling in the hippocampus that mediates the anti-neuroinflammatory as well as antiapoptotic effects, and finally, results in memory enhancing effects.

We further believe that the newly discovered binding affinity of curcumin to the mitochondrial enzyme 17-β-hydroxysteroid dehydrogenase type 10 (17β-HSD10) may open a new therapeutic avenue for treating AD. According to the literature, the brain of individuals with AD [102] as well as of animals in an AD mouse model [103] exhibits abnormally elevated levels of 17β-HSD10, which lead to the impairment of structure, function, and dynamics of mitochondria. This may underlie the pathogenesis of the synaptic and neuronal deficiency exhibited in 17β-HSD10 related diseases, including the AD [54].

Using our newly developed inverse molecular docking protocol, it is possible to predict binding modes and binding affinities of ligands with reasonable accuracy. However, the obtained docking scores do not directly correspond to binding free energies, which could be calculated using more advanced molecular dynamics simulation techniques [104,105].

## 4. Conclusions

In this paper, we used a novel inverse molecular docking protocol and as the first predicted potential targets of curcumin among all human proteins from the Protein Data Bank. We identified inhibitory effects of curcumin on numerous signaling pathways involved in carcinogenesis and tumor formation. We also found that curcumin potentially binds to proteins playing an important role in cognitive impairment associated with Alzheimer’s disease. Moreover, our results confirm some already suggested, and predict several new, molecular mechanisms responsible for curcumin’s efficacy as a chemopreventive and anti-inflammatory agent as well as clarify its value as a therapeutic drug. The obtained results will, therefore, direct future experiments by narrowing down the potential protein targets of curcumin.

The main obstacle to utilize curcumin as a chemopreventive therapeutic agent in humans remains its limited bioavailability, as well as its chemical instability at physiological conditions. We suggest that properly formulated delivery systems, such as lipid vesicles [98], nanoparticles [96,97] and nanofibers [106], might be able to boost the bioavailability and stability of curcumin. Moreover, scientists all over the world are investigating a number of different curcumin analogs that may be better absorbed and even more effective [107,108] through procedures that can be very demanding, expensive and time consuming. We firmly believe that our novel inverse molecular docking protocol represents also a promising opportunity to identify the most potent curcumin analogs and to consequently reduce the associated research costs.

All in all, the proposed inverse molecular docking protocol provides a promising opportunity to identify potential protein targets for other chemopreventive compounds originating from various natural sources as well as to predict the potential toxic side-effects stemming from interactions with proteins other than the targeted one in the drug-development process.

## Figures and Tables

**Table 1:** Identified curcumin’s protein targets using the developed inverse molecular docking protocol against human proteins.

PDB ID with Chain	Protein Name	Predicted Docking Scores (arb. Units)	Protein Function and Reported Connection with Diseases	Reported Experimental Correlation with Curcumin *
4kmyA	human folate receptor β (FR-β)	−63.30	A target for the specific delivery of antifolates or folate conjugates to tumors or sites of inflammation [49].	Yes [50]
3iadA	cAMP-specific 3′,5′-cyclic phosphodiesterase 4D (PDE4D)	−62.24	Modulation of cAMP signaling, important in the treatment of Alzheimer’s disease, Huntington’s disease, schizophrenia, and depression [51].	Yes [52]
1u7tA	17-β-hydroxysteroid dehydrogenase type 10 (17β-HSD10)	−61.46	Interacts with amyloid-β, connection with neuronal dysfunction associated with Alzheimer’s disease [53,54].	No
2qrvA	DNA (cytosine-5)-methyltransferase 3A	−58.59	Required for genome-wide de novo methylation of DNA. Represses transcription through HDAC [55].	Yes [56]
1ck7A	metalloproteinase-2 (MMP-2)	−57.93	Involved in angiogenesis, tissue repair, tumor invasion and inflammation. Initiates a primary innate immune response with the activation of the NF-κB transcriptional pathway [57,58].	Yes [59]
3qeoA	deoxycytidine kinase (dCK)	−57.37	Required for the phosphorylation of deoxyribonucleosides and nucleoside analogs in antiviral and chemotherapeutic agents [60].	No
4x3oA	NAD-dependent protein deacetylase sirtuin-2	−56.96	Involved in the cell cycle, genomic integrity, microtubule dynamics, cell differentiation, metabolic networks, and autophagy. Deacetylates RELA in the cytoplasm inhibiting NF-κB-dependent transcription activation upon TNF-α stimulation [61].	No
3e7oA	mitogen-activated protein kinase 9 (MAPK-9)	−56.93	Regulates cell proliferation, differentiation, migration and programmed cell death. Phosphorylates AP-1 components c-Jun and ATF2 and thus regulates AP-1 transcriptional activity. Promotes β-catenin/CTNNB1 degradation and inhibits the Wnt signaling pathway [62,63].	Yes [64]
4h2iA	ecto-5′-nucleotidase (e5NT)	−55.95	Activates P1 adenosine receptors, and has emerged as a drug target for treatment of inflammation, chronic pain, hypoxia, and cancer [65].	No
4nwgA	tyrosine-protein phosphatase non-receptor type 11	−55.49	Positively regulates the MAPK signal transduction pathway [66].	No
1zr3A	core histone macro-H2A.1	−55.46	Inhibits histone acetylation by EP300, recruits class I HDACs, which represses transcription. Inhibits the binding of transcription factor NF-κB [67,68].	No
4zzjA	NAD-dependent protein deacetylase sirtuin-1	−54.89	Coordinates the cell cycle, response to DNA damage, metabolism, apoptosis, deacetylation of histones and autophagy. Deacetylates ‘Lys-382’ of p53/TP53 as well as RELA/NF-κB p65 and impairs its ability to induce apoptosis. Modulates AP-1 transcription factor activity [69,70,71].	No
4zseA	epidermal growth factor receptor	−54.81	Activates major downstream signaling cascades Ras-RAF-MEK-ERK, PI3 kinase-AKT, PLCγ-PKC, STATs modules and NF-κB. [72,73]	Yes [74]
5kviA	apoptosis-inducing factor 1 (AP-1)	−54.76	NADH oxidoreductase and a regulator of apoptosis in a caspase-independent pathway [75].	Yes [76]
3lcoA	macrophage colony-stimulating factor 1 receptor (CSF1R)	−54.59	Regulates proliferation and differentiation of macrophages and monocytes. Promotes the release of proinflammatory chemokines in response to IL34 and CSF1. Mediates activation of the MAPK1/ERK2 and/or MAPK3/ERK1 [77,78].	No
2rgcA	GTPase HRas	−54.43	Activation of Ras signal transduction pathway [79].	No
2clpA	aflatoxin B1 aldehyde reductase member 3	−53.86	Reduces the dialdehyde protein-binding form of aflatoxin B1 (AFB1) to the non-binding AFB1 dialcohol. Involved in the protection of the liver against the toxic and carcinogenic effects of AFB1 [80].	No
1s1pA	aldo-keto reductase family 1 member C3 (AKR1C3)	−53.69	Suppresses cell differentiation and promotes proliferation in myeloid cells. Possesses potential in new anticancer therapies with reduced COX-dependent side effects [81].	No
3hi7A	amiloride-sensitive amine oxidase	−53.51	Catalyzes cell proliferation, tissue differentiation, tumor formation, and possibly apoptosis [82].	No
2a2aA	death-associated protein kinase 2	−53.41	Triggers cell survival, apoptosis, and autophagy. Regulates type I apoptotic and type II autophagic cell death signals, depending on the cellular setting [83]	No
1r6tA	tryptophan-tRNA ligase	−53.31	Regulates ERK, AKT (PKB), and eNOS activation pathways associated with angiogenesis [84].	No

* Yes—known interaction between the curcumin and the protein, not necessarily implying direct binding of curcumin to the active site; No—no known interaction with curcumin.

## Supplementary Materials

The following are available online. Table S1: Details for FR-β-curcumin interactions identified by PLIP. Table S2: Details for PDE4D-curcumin interactions identified by PLIP.

## Abbreviations

ABAD	amyloid β-peptide-binding alcohol dehydrogenase
AD	Alzheimer’s disease
AFB1	aflatoxin B1
AKR1C3	aldo-keto reductase family 1 member C3
AKT	protein kinase B (PKB)
AP-1	activator protein 1
ATF2	activating transcription factor 2
cAMP	cyclic adenosine monophosphate
COX	cyclooxygenase
CSF1	the colony stimulating factor 1
CSF1R	macrophage colony-stimulating factor 1 receptor
dCK	deoxycytidine kinase
DNA	deoxyribonucleic acid
e5NT	ecto-5’-nucleotidase
eNOS	endothelial nitric oxide synthase 3
ERK	extracellular signal-regulated kinases
EP300	histone acetyltransferase p300
FADD	fas-associated protein with death domain
FR-β	human folate receptor β
HATs	histone acetyltransferases
HDACs	histone deacetylases
HSD10	17-β-hydroxysteroid dehydrogenase type 10
IL-1	interleukin 1
IκB	inhibitory protein kappa B
MMPs	matrix metalloproteinases
MAPK	mitogen-activated protein kinases
MEK	mitogen-activated protein kinase kinase
NF-κB	nuclear factor κB
NMR	nuclear magnetic resonance
nr-PDB	non-redundant Protein Data Bank
PKC	protein kinase C
PDB	Protein Data Bank
PDEs	cyclic nucleotide phosphodiesterase enzymes
PI3	phosphatidylinositide 3-kinase
PLCγ	phospholipase C gamma
RAF	rapidly accelerated fibrosarcoma
ROS	reactive oxygen species
STAT	signal transducer and activator of transcription protein
TNF-α	tumor necrosis factor α
TP53	tumor protein p53
tRNA	transport ribonucleic acid
Wnt	wingless-related integration site
