# Supplementary material for: Inverse Molecular Docking as a Novel Approach to Study Anticarcinogenic and Anti-Neuroinflammatory Effects of Curcumin
Source: Molecules. 2018 Dec 18;23(12):3351. doi: 10.3390/molecules23123351 (PMC6321024; doi:10.3390/molecules23123351)
Supplement: Supplementary file 1 [file molecules-23-03351-s001.pdf]

Supplementary material

Inverse Molecular Docking as a Novel Approach to Study Anticarcinogenic and Anti-Neuroinflammatory Effects of Curcumin

Veronika Furlan,[1] Janez Konc,[*,2] Urban Bren[*,1, 2]

[1]Faculty of Chemistry and Chemical Technology, University of Maribor, Smetanova 17, SI-2000 Maribor, Slovenia; veronika.furlan@um.si

[2]National Institute of Chemistry, Hajdrihova 19, SI-1000, Ljubljana

*Correspondence: janez.konc@ki.si; Phone: +386-1-476 0273; urban.bren@um.si; Phone: +386-2-229 4421.

Table S1. Details for FR-β-curcumin interactions identified by PLIP.

Hydrogen bonds			
Residue	Distance D-H--A (Å)	Angle (°)	H-bond donor?
APS 97	0.88	155.72	No
HIS 151	0.82	145.33	No
ARG 152	0.78	136.90	Yes
ARG 152	0.68	129.84	No
SER 190	0.95	169.07	Yes
π-Stacking interactions			
Residue	Distance (Å)	Angle (°)	Type
TYR 101	4.32	4.35	P
TRP 187	3.96	28.59	P
TRP 187	4.12	27.53	P

*D – donor, A – acceptor, P - parallel.

Table S2. Details for PDE4D-curcumin interactions identified by PLIP.

Hydrogen bonds			
Residue	Distance D-H--A (Å)	Angle (°)	H-bond donor?
HIS 366	0.91	162	No
ASN 375	0.63	120.95	Yes
MET 439	0.81	140.25	Yes
ASN 602	0.73	131.99	Yes
Hydrophobic interactions			
Residue	Distance (Å)	Angle (°)	Type
MET 439	3.61	NA	NA

*D – donor, A – acceptor, NA – not applicable .
